# *Mycobacterium mucogenicum* bacteremia: major role of clinical microbiologists

**DOI:** 10.1186/s12879-018-3545-3

**Published:** 2018-12-12

**Authors:** Maxime Pradier, Anne Boucher, Olivier Robineau, Elisabeth Chachaty, Agnès Meybeck, Eric Senneville

**Affiliations:** 1 Service Universitaire des Maladies Infectieuses et du voyageur, Centre Hospitalier Dron, 135 avenue du Président Coty, 59200 Tourcoing, France; 2 Laboratoire de Microbiologie, Institut Gustave Roussy, Villejuif, France

**Keywords:** *Mycobacterium mucogenicum*, Rapidly growing mycobacteria, Bacteremia, Catheter-related infection

## Abstract

**Introduction:**

*Mycobacterium mucogenicum* is a rare but emerging cause of infections, especially in immunocompromised patients.

**Case presentation:**

We describe a new case of *M. mucogenicum* catheter-related bloodstream infection in a 34-year-old woman with ovarian cancer. *M. mucogenicum* was at first considered as a contaminant, and susceptibility testing was not performed. Usual susceptibility of *M. mucogenicum* motivated prescription of clarithromycin and moxifloxacin. Finally, our isolate was confirmed susceptible to both drugs. Clinical outcome was favorable with no relapse of infection after antibiotics discontinuation despite concomitant chemotherapy.

**Conclusion:**

Our case illustrates the need for a clinician-microbiologist dialogue in case of suspected *M. mucogenicum* infection to avoid delaying appropriate management.

## Background

Non-tuberculous mycobacteria are an important cause of human infections. Among them, rapidly growing mycobacteria are defined by their ability to produce mature colonies on agar plates within 7 days. They are rare but recently increasing causes of infections, especially in immunocompromised patients [1]. One of the most common form of disease is catheter-related bloodstream infection [2]. The pathogen most frequently implicated in case of catheter-related bloodstream is *Mycobacterium mucogenicum* [3]. Because rapidly growing mycobacteria are ubiquitous environmental microorganisms, their isolation requires a careful assessment of their clinical significance. We report a new case of *M. mucogenicum* bacteremia and discuss the role of microbiologists and its clinical management.

## Case presentation

A 34-year-old woman with platin-resistant metastatic ovarian cancer experimented fever and chills during intravenous infusion of the anti-PD-1 antibody, nivolumab. The patient’s temperature was 39.5 °C, pulse rate was 120/min, and blood pressure was 95/60. Physical examination showed nausea and a known pelvic mass. The cardiopulmonary auscultation was normal. There were neither cutaneous nor mucous abnormalities. Allergic reaction was suspected. Infusion was stopped, antihistamine and corticosteroids (1 mg/kg of methylprednisolone) were administered. The patient remained febrile. Gram stain of a positive blood culture revealed bacillus Gram-positive bacteria. Combination therapy with amoxicillin and gentamicin was started. Finally, three blood cultures, one drawn from the port-a-cath and two from a peripheral vein returned positive for rapidly growing mycobacteria. The port-a-cath was removed and the antibiotics were switched to gentamicin, clarithromycin, and ethambutol. Transthoracic echocardiography revealed no sign of endocarditis. The isolate was sent to a local referral laboratory for identification and susceptibility testing. Identification to the species level was performed by Matrix-assisted laser desorption/ionization time-of-flight mass spectrometry (MALDI-TOF mass spectrometry) (Vitek MS, BioMerieux®, Marcy l’Etoile, France), leading to *Mycobacterium mucogenicum* identification. In the absence of clinical information, *M. mucogenicum* was considered as a contaminant, and susceptibility testing was not performed. Nevertheless, diagnosis of *M. mucogenicum* port-a-cath related bloodstream infection was retained. Two weeks after starting antibiotic treatment, renal impairment urged its change. Usual susceptibility of *M. mucogenicum* motivated switch to clarithromycin and moxifloxacin. Our isolate was sent to the National Consultant Laboratory for Mycobacteria for sensitivity testing. Identification was confirmed through sequencing analysis of hsp65 gene. Susceptibility testing was performed by standard broth microdilution method using Sensititre® RAPMYCO panel. The isolate was susceptible to clarithromycin and moxifloxacin (Table 1). The occurrence of this infection prevented the poursuit of experimental infusion of nivolumab. After 2 months of combination antibiotherapy, cyclophosphamide was started because of worsening of peritoneal carcinomatosis. Antibiotics were pursued 6 months. No relapse of infection was observed after its discontinuation despite concomitant chemotherapy.

**Table 1:** Susceptibility testing of *Mycobacterium mucogenicum* isolate

Antimicrobial drugs	Minimal inhibitory concentration (mg/L)	Clinical breakpoints^*^ (mg/L)
amikacin	2	16–64
tobramycin	8	2–8
ciprofloxacin	0.5	1–4
moxifloxacin	< 0.25	1–4
clarithromycin	0.5	2–8
linezolid	2	4–32
trimetoprim/sulfamethoxazole	< 0.25/4.75	2/38–4/76
cefoxitin	< 4	16–128
imipenem	< 2	4–32
doxycyclin	0.25	1–8
minocyclin	< 1	1–8

^*^Clinical breakpoints according to the European Committee on Antimicrobial Susceptibility Testing (EUCAST)

## Discussion

We report a new case of *Mycobacterium mucogenicum* catheter related bloodstream infection, occuring in an immunocompromised patient. *M. mucogenicum* belongs to the group of rapidly growing mycobacteria, which are ubiquitous environmental organisms. Previously known as *M. chelonae*-like organism, *M. mucogenicum* finally changed name because of its phylogenetic distance from *M. chelonae*, but closeness to *M. fortuitum* and because of its mucoid colonies [4].

Infections caused by rapidly growing mycobacteria have been increasingly reported during the past few years because of improvement of isolation and identification techniques and spread of medical conditions compromising immune system [1, 5]. These microorganisms have been shown to cause various infections, including bacteremia. *M. mucogenicum* is the most common rapidly growing mycobacteria implicated in catheter related bloodstream infections [2, 3]. Like other rapidly growing mycobacteria, it has a high predisposition to create biofilm and colonise intravascular devises. Isolates appear as Gram-positive bacteria on Gram stain. Acid-fast stain is positive. *M. mucogenicum* can be cultivated in Lowenstein-agar but also in routine culture media within 7 days. The current gold standard for the identification of mycobacteria is DNA sequencing with 16sRNA gene, *rpo*B, and hsp65 being recognized as useful targets [6, 7]. But these methods are not affordable in many laboratories. Several investigators have demonstrated that MALDI-TOF mass spectrometry could accurately identify mycobacteria [8, 9]. Since treatment and response rates differ widely depending on the mycobacterial species, rapid identification is essential. Prompt identification to the species level can predict in vitro susceptibility and guide the choice of initial antibiotic therapy. Despite the possibility of contamination, recovery of *M. mucogenicum* from the bloodstream especially in immune-compromised patients should be considered as a true pathogen. Susceptibility testing is indicated for any rapidly growing mycobacteria considered clinically significant. *M. mucogenicum* isolates are usually susceptible to aminoglycosides, fluoroquinolones, tetracyclines, macrolides, carbapenems, cefoxitin, trimethoprim-sulfamethoxazole, and linezolid [10]. Management of *M. mucogenicum* catheter-related bloodstream infections is mainly based on clinical experience. Optimal antibiotherapy is not established. In previously published case-series, an aminoglycoside combined with a macrolide and/or a quinolone was the most common empirical treatment [5, 11]. Optimal duration of treatment is unknown. At least 4 weeks of combination regimen were prescribed. But treatment may be prolonged in case of deep and persistent immunosuppression. Removal of the catheter is required to achieve successful outcome. Indeed, relapses have been associated with preservation of the catheter [3]. The mortality rate was usually low [3, 11].

## Conclusion

*M. mucogenicum* is an emerging cause of catheter related bloodstream infection. Awareness of clinicians and microbiologists should be raised to avoid considering *M. mucogenicum* as a contaminant and delaying initiation of antibiotherapy. Absence of established therapeutic guidelines makes case reports relevant to increase data on various antibiotic regimen and prognosis.

## Abbreviations

DNA: Deoxyribonucleic acid
MALDI-TOF: Matrix-assisted laser desorption/ionization time-of-flight mass spectrometry
RNA: Ribonucleic acid
